# Anatomical Study of the Inferior Oblique Muscle and Its Innervation: Morphometric Characteristics, Anatomical Variations, and Histological Evaluation of the Nerve to the Inferior Oblique Muscle

**DOI:** 10.3390/brainsci14090925

**Published:** 2024-09-17

**Authors:** Robert Haładaj, R. Shane Tubbs, Ivan Varga

**Affiliations:** 1 Institute of Histology and Embryology, Faculty of Medicine, Comenius University in Bratislava, 81372 Bratislava, Slovakia; ivan.varga@fmed.uniba.sk; 2 Department of Neurosurgery, Tulane University School of Medicine, New Orleans, LA 70112, USA; shane.tubbs@icloud.com; 3 Department of Neurology, Tulane University School of Medicine, New Orleans, LA 70112, USA; 4 Department of Structural & Cellular Biology, Tulane University School of Medicine, New Orleans, LA 70112, USA; 5 Department of Surgery, Tulane University School of Medicine, New Orleans, LA 70112, USA; 6 Department of Anatomical Sciences, St. George’s University, St. George 1473, Grenada; 7 Department of Neurosurgery and Ochsner Neuroscience Institute, Ochsner Health System, New Orleans, LA 70433, USA; 8 Brisbane Clinical Neuroscience Centre, University of Queensland, Brisbane, QLD 4072, Australia

**Keywords:** anatomical variations, inferior oblique muscle, nerve to the inferior oblique muscle, oculomotor nerve, orbit

## Abstract

Background: This report aims to supplement the existing knowledge on the inferior oblique muscle. In particular, this study presents detailed anatomical and histological data concerning the muscle’s entry point (or entry zone) of the nerve to the inferior oblique muscle. Particular attention was paid to the topographical relationships of the nerve to the inferior oblique muscle (NTIO), including the location of its entry point to the muscle’s belly and its anatomical variations. Methods: Sixty orbits from cadaveric hemi-heads fixed in 10% formalin were studied. The course of the NTIO was traced along the lateral border of the inferior rectus muscle as far as its entry point to the inferior oblique muscle. Particular attention was paid to the various ways in which the NTIO’s muscular sub-branches penetrated between the fibers of the inferior oblique muscle. Results: Three types of NTIO entries to the inferior oblique muscle’s belly were distinguished. In the most common type (48.3%), the nerve entered the muscle’s inferior (orbital) surface. In the next most common type (36.7%), terminal muscular sub-branches of the NTIO joined the superior (also referred to as ocular or global) surface of the inferior oblique muscle. In the remaining four cases (15%), the terminal sub-branches of the NTIO were divided into two main groups (superior and inferior) that joined both the superior and inferior surfaces of the muscle. Histological examination confirmed that the distal part of the NTIO shows a characteristic arcuate course (angulation) just before reaching the muscle’s belly. The process for splitting and forming separate muscular sub-branches of the NTIO was observed for all the examined histological specimens at the level of the nerve’s angulation. Conclusions: The presented findings enhance the understanding of the anatomical variations and precise distribution of motor sub-branches reaching the inferior oblique muscle, which may deepen anatomical knowledge and potentially enhance the management of ocular motor disorders.

## 1. Introduction

Most extraocular muscles originate in the common tendinous ring (annulus of Zinn) and run divergently forward, attaching to the eyeball via tendinous strands. The exception is the inferior oblique muscle, which originates in the distal part of the inferior orbital wall (on the orbital surface of the maxilla), surrounds the eyeball from below (covering the external surface of the inferior rectus muscle’s insertion), and attaches to the scleral external surface (on the eyeball’s posterior, inferolateral aspect), deep to the tendon of the lateral rectus muscle [1]. The primary action of this muscle is extortion (external rotation) of the eyeball, but it also produces eyeball elevation and abduction. A branch of the inferior division of the oculomotor nerve provides its motor innervation. This branch is called the nerve to the inferior oblique muscle (NTIO) [2,3,4,5].

Extraocular muscles and the nerves supplying them can be injured during traumas or intraorbital medical procedures. The inferior oblique muscle is located near the anterior orbital margin, together with the NTIO that supplies it. This anatomical relationship should be remembered when diagnosing orbital traumas and surgical accesses and procedures within the inferior orbit [5,6,7]. Among the papers on the innervation of extraocular muscles, only a few reports have detailed the innervation of the inferior oblique muscle [3,8,9,10,11,12,13]. Understanding the histology of the nerve to the inferior oblique muscle may be helpful in various clinical contexts, such as diagnosing nerve damage or neuropathies that can affect eye movement.

Our report aims to supplement the anatomical description of the inferior oblique muscle with additional data. Particular attention was paid to the topographical relationships of the NTIO, the location of its entry point (or entry zone) to the inferior oblique muscle’s belly, and the anatomical variations of its terminal sub-branches. Concerning the angulation of the muscle’s entry point of the nerve to the inferior oblique muscle, which is not described morphologically enough, new data, based on histological analysis, are presented. The available literature was also analyzed.

## 2. Materials and Methods

This study was conducted on 60 orbits (29 right and 31 left) taken from cadaveric hemi-heads fixed in 10% buffered formalin (all the cadavers were Central European). The exclusion criteria were craniofacial malformations, scars, or traces of previous surgical interventions within the heads. This study complied with the Declaration of Helsinki.

The dissection was performed following previously adopted microanatomical protocols. The superior and lateral orbital walls were removed, exposing the superior orbital fissure and the optic canal [14,15]. The periorbit was separated from the remaining bony boundaries of the orbit using blunt dissection; the nerves and vessels were mobilized and cut, and the origin of the inferior oblique muscle was gently separated. All the orbital content was then carefully removed. The NTIO was traced and cleaned from its origin to its junction among the fibers of the inferior oblique muscle. The course of the nerve to the inferior oblique muscle was traced along the lateral border of the inferior rectus, as far as the point of entry of the neurovascular bundle to the inferior oblique muscle. At this stage of the dissection, particular attention was paid to the various ways in which this nerve penetrated between the fibers of the inferior oblique muscle. A chi-squared calculator (Social Sciences Statistics) was used to evaluate differences in anatomical variations by sides.

**Figure 1:**
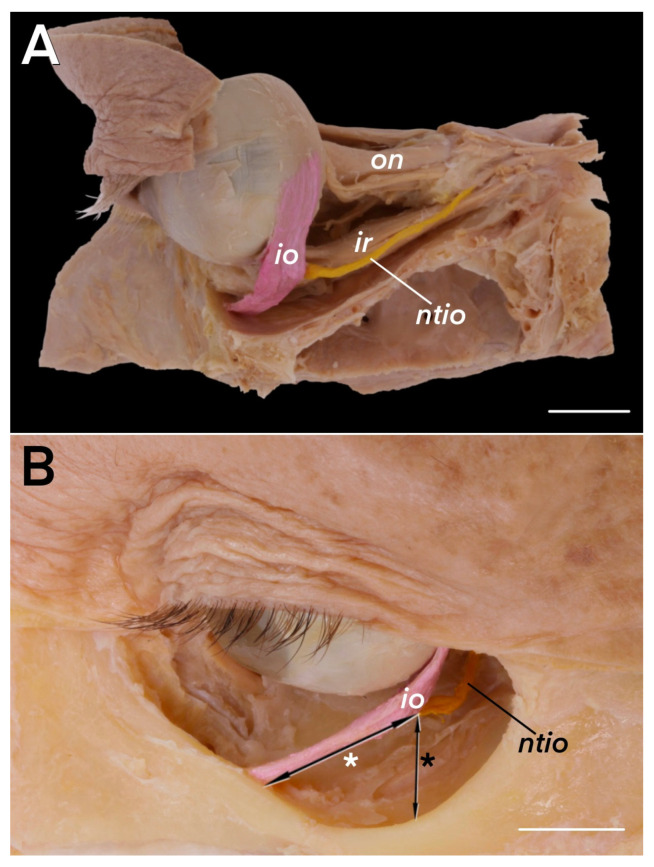
Anatomical relationships of the inferior oblique muscle (io; colored pink) and its motor innervation (ntio; colored yellow). (**A**) Lateral view of the left orbit. The nerve to the inferior oblique muscle is exposed in its course along the lateral border of the inferior rectus muscle (ir). The scale bar shows 10 mm. (**B**) Antero-lateral view of the right orbit. The origin of the inferior oblique muscle is exposed. The distance between the muscle’s origin and the entry point of its motor branch (ntio) is marked by a black line with a double arrowhead and a white asterisk. The vertical distance between the inferior orbital rim and the nerve to the inferior oblique muscle’s entry point to the inferior oblique muscle is marked by a black line with a double arrowhead and a black asterisk. The scale bars show 10 mm; on—optic nerve.

An electronic caliper was used to measure the length and diameter of the muscular branch innervating the inferior oblique muscle, the length of that muscle, the width of its attachments, the thickness of its origin, and the distance between the origin (Figure 1B) and the point of entry of the motor branch innervating the muscle. The vertical distance between the inferior orbital margin and the point of entry of the NTIO was also measured. Basic descriptive statistics were used to structure the data.

Ten inferior oblique muscles were harvested with the terminal part of the NTIO. The specimens were subjected to histological examination using the classical paraffin technique [16,17] and HE staining to expose the NTIO’s entry point (entry zone) to the muscle’s belly (the junction between the NTIO’s motor subdivisions and the muscle’s belly) in detail.

## 3. Results

### 3.1. Morphometric Analysis of the Inferior Oblique Muscle and Its Motor Branch

The inferior oblique muscle showed typical location and morphology in all the specimens examined. The mean muscle length was 31.2 mm (S.D. = 1.72 mm). The mean width of the origin was 3.29 mm (S.D. = 0.58 mm), while the mean width of the insertion was 7.76 mm (S.D. = 0.79 mm). Detailed measurements of the inferior oblique muscle are presented in Table 1.

The mean length of the NTIO was 30.6 mm (S.D. = 3.41 mm), and its mean diameter was 0.82 mm (S.D. = 0.12 mm). Its detailed measurements are presented in Table 1. The vertical distance between the neurovascular bundle’s (including the NTIO’s) entry point to the posterior border of the inferior oblique muscle and the inferior orbital margin was 3.66 mm (S.D. = 0.93; Figure 1B, Table 1). The mean distance between the inferior oblique muscle and the neurovascular bundle to the muscle’s belly was 15.23 mm (median = 15.06, S.D. = 2.09 mm; Figure 1B, Table 1).

### 3.2. Anatomical Study Results

**Figure 2:**
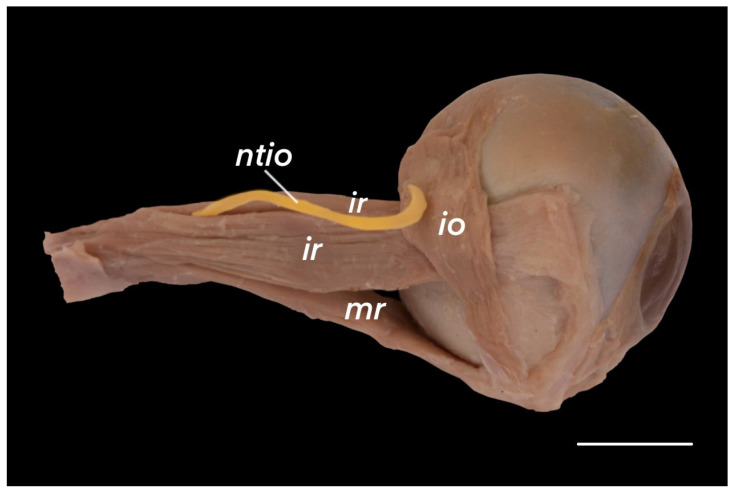
The variant in which the nerve to the inferior oblique muscle (ntio) pierces the inferior rectus muscle (ir). Inferior view of the right orbit. In this variant, the ntio emerges from the inferior rectus muscle’s belly within the proximal half of the muscle’s length. The scale bars show 10 mm; io—inferior oblique muscle; mr—medial rectus muscle.

**Figure 3:**
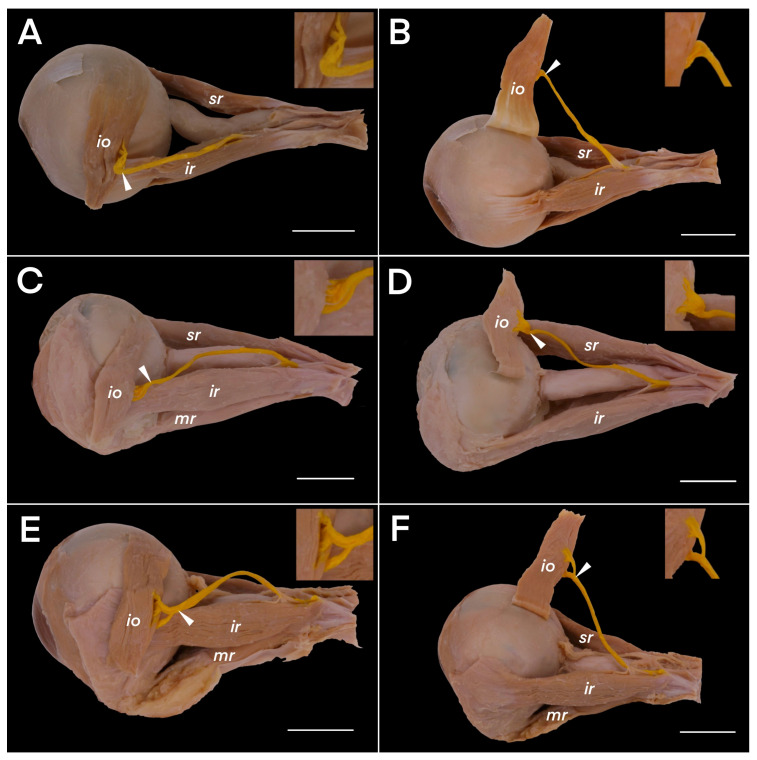
Three types of nerves to the inferior oblique muscle’s entry points (marked by white arrowheads) to the muscle’s belly (io). The motor branch, i.e., the nerve to the inferior oblique muscle, is colored yellow. (**A**) The most common type (48% of the cases). The nerve to the inferior oblique muscle enters the muscle’s inferior (orbital) surface in this type. (**B**) The same specimen as in (**A**), with the inferior oblique muscle reflected to show the absence of the nerve’s sub-branches on the muscle’s superior (also referred to as ocular or global) surface. (**C**) The next most common type (36% of the cases) in which terminal muscular sub-branches join the superior (ocular) surface of the inferior oblique muscle. No motor sub-branches are visible on the inferior (orbital) surface of the muscle. (**D**) The same specimen as in (**C**), with the inferior oblique muscle reflected to show the nerve’s sub-branches on the muscle’s superior (ocular or global) surface. (**E**) Another variant (16% of the cases), in which the terminal sub-branches of the NTIO cover the muscle’s posterior border and form two main subdivisions (superior and inferior) that join both the superior (ocular) and inferior (orbital) surfaces of the muscle. This figure shows an inferior group of sub-branches joining the inferior surface of the inferior oblique muscle. (**F**) The same specimen as in (**E**), with the inferior oblique muscle reflected to show the superior group of nerve sub-branches joining the muscle’s superior surface. The scale bars show 10 mm; ir—inferior rectus muscle; mr—medial rectus muscle; sr—superior rectus muscle.

In all the cases, the NTIO, i.e., the motor branch supplying the inferior oblique muscle, originated from the inferior branch (inferior division) of the oculomotor nerve and initially ran on the superolateral surface of the inferior rectus muscle (Figure 1A). It then traveled inferolaterally along the lateral border of the inferior rectus muscle to innervate its target, the inferior oblique muscle (Figure 1A). In 85% of the cases (51/60), the parasympathetic root of the ciliary ganglion (branch to the ciliary ganglion) originated from the NTIO. In nine of the 60 specimens (15%; four right and five left), the NTIO pierced the inferior rectus muscle (Figure 2 and Figure 3A,B) instead of running along its lateral border, as in the most common arrangement. In those nine cases, the NTIO was surrounded by inferior rectus muscle fibers, creating a subtle, cleft-like separation of the fibers, and emerged from the inferior rectus muscle’s belly in the proximal half of its length. When the NTIO pierces the inferior rectus muscle, the muscular branches to the inferior rectus typically originate from the inferior division of the oculomotor nerve before the NTIO enters the inferior rectus fibers.

Before entering the inferior oblique muscle, the NTIO’s distal part created a characteristic sharp angulation, so the nerve fibers reached the muscle’s belly at a nearly right angle to the NTIO’s long axis. There were three types of NTIO’s entry zones to the muscle’s belly (junction between the NTIO’s motor subdivisions and the muscle’s belly). In the most common type (29 of the 61 specimens, i.e., 48.3%, on 13 right and 16 left sides), the nerve entered the muscle’s orbital (inferior) surface. In this type, no muscular sub-branches reached the muscle’s superior (also referred to as ocular or global) surface (Figure 3A,B). In the next most common type (22 of the 60 cases, i.e., 36,7%, on 12 right and 10 left sides), the terminal muscular sub-branches of the NTIO joined the ocular surface of the muscle (Figure 3C,D). In the remaining nine cases (9/60, i.e., 15%, four right and five left sides), the terminal sub-branches of the NTIO extended superiorly and inferiorly to encompass the posterior border of the muscle. They formed two main subdivisions (superior and inferior) that joined both the superior and inferior surfaces (Figure 3E,F). There were no statistically significant differences in the incidences of each type of NTIO entry by side (the chi-squared statistic was 0.5372, and the *p*-value was 0.7644; the result is not significant at *p* < 0.05).

### 3.3. Histological Examination

**Figure 4:**
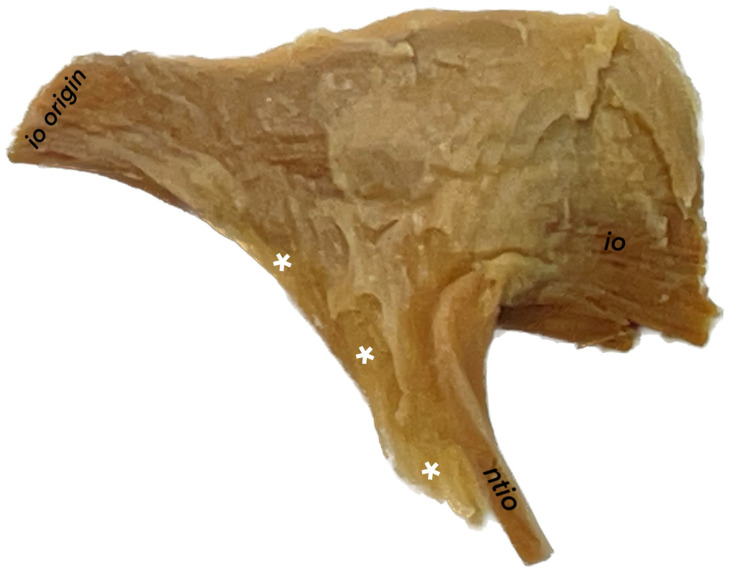
Specimen of the inferior oblique muscle (io) before paraffin embedding. The inferior (orbital) surface of the muscle is exposed. The distal fragment of the neurovascular bundle, including the nerve to the inferior oblique muscle (ntio), is visible, along with the fascia and connective tissue fragment (marked by white asterisks) that connects the inferior oblique muscle to the inferior rectus muscle, acting as an ancillary origin of the inferior oblique muscle and functioning as a muscle pulley. The distal part of the ntio is only topographically related to this fascial connection between the inferior oblique and inferior rectus muscles.

**Figure 5:**
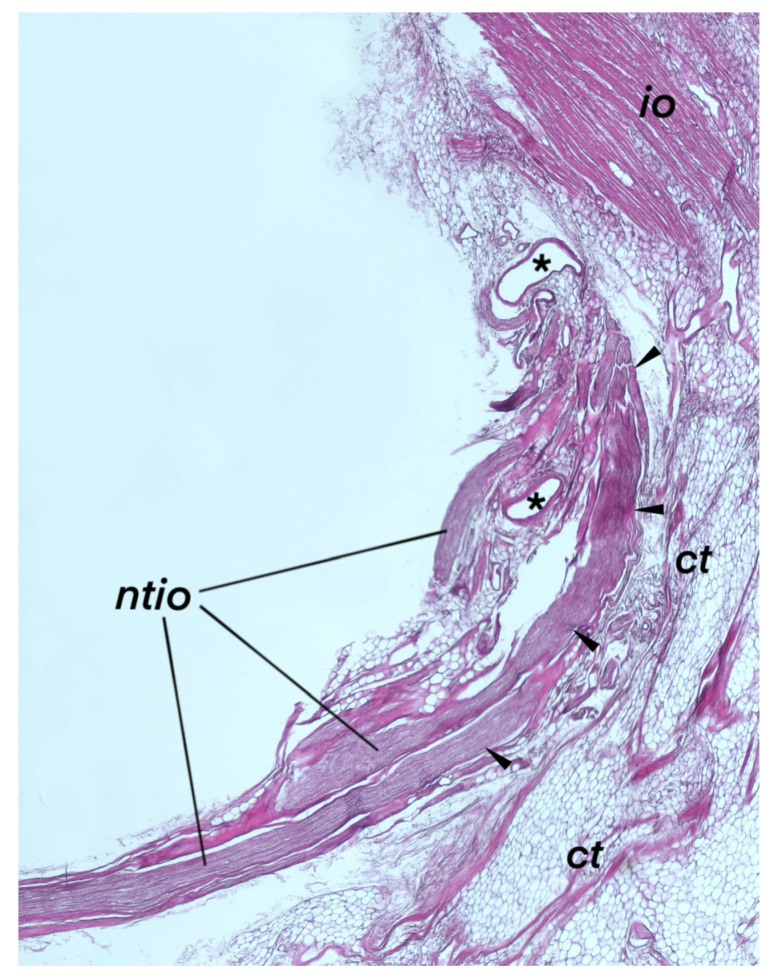
Histological specimen showing the terminal part of the nerve to the inferior oblique muscle (ntio). HE staining. The distal part of the ntio shows a characteristic curved course (marked by black arrowheads) and enters the border of the inferior oblique muscle (io) toward the muscle’s origin. Blood vessels (marked by black asterisks) accompany the nerve. The entire neurovascular bundle containing the ntio and blood vessels is covered by connective and adipose tissues (ct). The epineurium (the outermost nerve’s covering, consisting of dense, irregular connective tissue) and perineurium (a sheath of specialized connective tissue that encloses each nerve’s fascicle) are visible at the nerve’s angulation level.

**Figure 6:**
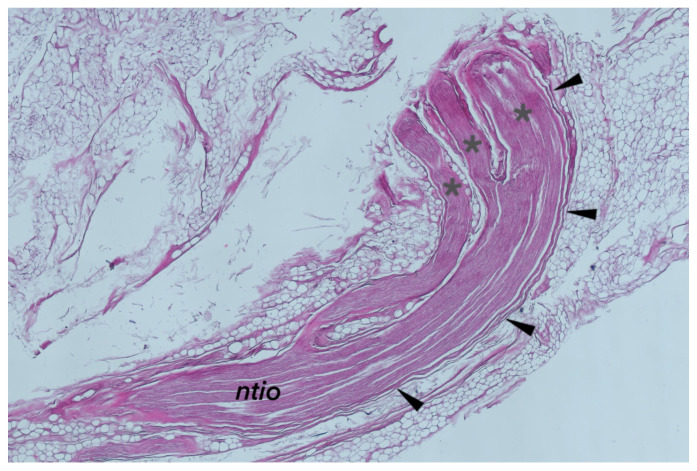
The histological examination of the nerve to the inferior oblique muscle (ntio). The nerve’s distal part course shows a characteristic angulation (marked by black arrowheads). At the angulation level, the nerve is divided into motor subdivisions (marked by gray asterisks) that enter the muscle’s belly separately. The presence of the accompanying connective tissue may also be noted, emphasizing the complexity of the neurovascular bundle.

Histological examination enabled more detailed observations of the interface of the neurovascular bundle and the posterior border of the inferior oblique muscle. Figure 4 shows a specimen of the inferior oblique muscle before paraffin embedding. The histology of the nerve innervating the inferior oblique muscle, specifically, the NTIO, exhibited distinct features typical of peripheral motor nerves. Histological specimens showing the terminal part of the NTIO are shown in Figure 5 and Figure 6. Histological examination confirmed that the distal part of the NTIO shows a characteristic arcuate course (angulation) just before reaching the muscle’s belly; i.e., it enters the border of the inferior oblique muscle at an angle to the NTIO’s long axis. It penetrates the muscle’s border and then sends muscular sub-branches toward the muscle’s origin (Figure 5 and Figure 6). The NTIO’s entry to the muscle’s belly was split into motor subdivisions at the angulation level so that individual muscular sub-branches entered the muscle’s belly separately (Figure 6). The process for splitting and forming separate muscular sub-branches of the NTIO was, therefore, observed in all the examined histological specimens at the level of the nerve’s angulation. Figure 6 shows a clear division of the NTIO into motor sub-branches at the NTIO’s angulation point.

Blood vessels accompanied the NTIO, and connective and fatty tissues covered the entire neurovascular bundle joining the inferior oblique muscle (Figure 5 and Figure 6). Histologically, the NTIO’s neurovascular bundle lacks the mechanical properties needed to function as an ancillary origin of the inferior oblique muscle. The distal part of the NTIO is only topographically related to the fascial connection between the inferior oblique and inferior rectus muscles, which actually functions as a muscle pulley, providing additional (ancillary) stabilization and mechanical support.

## 4. Discussion

### 4.1. NTIO’s Course and Variations

According to Tsutsumi et al. [10], several topographical aspects the NTIO are structurally consistent. However, the course of the nerve makes it prone to injury along the lateral border of the inferior rectus muscle. It can also occasionally pierce the inferior rectus muscle [18]; the incidence of this variation has been estimated at 15.4% [19] or 20% [20], and 15% of the specimens examined in the present study had it. It was also reported by Bergman et al. [21], who noted that along the orbital segment of the oculomotor nerve to the inferior oblique muscle, some nerve branches can pierce the inferior rectus muscle or even the ciliary ganglion. Injury to the NTIO can cause inferior oblique muscle impairment and impair the parasympathetic fibers that typically course with it [6,15,22,23]. In most cases, the parasympathetic root of the ciliary ganglion originates from the nerve to the NTIO [4,15,19], as also observed in the present study. Erdogmus et al. [8] found that the mean diameter of the branch entering the inferior oblique muscle was 1.04 mm (S.D. = 0.29 mm, range 0.59–1.56 mm) on the right side and 1.07 mm (S.D. = 0.23 mm, range 0.72–1.56 mm) on the left. Our results were slightly lower, the NTIO’s mean diameter being 0.82 mm (DS = 0.12 mm, range 0.61–1.05 mm). However, it should be acknowledged that postmortem shrinkage can affect anatomical measurements, particularly in soft tissues, like muscles and nerves, potentially slightly altering their size and spatial relationships.

### 4.2. NTIO’s Entry Point (Entry Zone) to the Inferior Oblique Muscle

As mentioned in the introduction, several papers have discussed the innervation of the inferior oblique muscle. In one recent study, Shin et al. [12] examined the detailed distribution of motor sub-branches within the muscle using Sihler’s stain. They discovered that on reaching the inferior oblique muscle, the NTIO divides into multiple smaller branches without distinct subdivisions, forming a root-like arborization and supplying the entire muscle’s width. Shin et al. [12] emphasized that the oculomotor nerve enters the inferior oblique muscle around its mid-length, which is consistent with our results (Table 1). However, these authors did not link the types of NTIO entries to the muscle’s belly with the observed variants of the distribution of intramuscular motor subdivisions. In the literature searched, only Kumar et al. [9] provided detailed information about the inferior oblique muscle, with emphasis on its nerve entry (motor branch entry to the muscle’s belly). They found that the NTIO entered the inferior oblique muscle through the inferior (orbital) surface in 75% of the cases, through the superior (ocular) surface in 17.9%, and through the posterior border in 7.1%. The frequencies were slightly different in our sample. Like Kumar et al. [9], we found the most common type to be the nerve entering the muscle’s inferior (orbital) surface, but the incidence was lower than theirs (48.3%). In the next most common type (36.7%), the NTIO entered the ocular surface of the inferior oblique muscle; we found a higher incidence of this type than Kumar et al. [9] did. In the remaining cases (15%), the NTIO’s divisions reached both the superior (ocular) and inferior (orbital) surfaces of the muscle. The sample sizes were relatively small in both studies, which could explain the numerical differences. In contrast, Ana-Magadia [11] found the NTIO’s junction with the inferior oblique muscle on the inferior surface of the muscle in all the orbits. Examining our specimens alongside those photographed by Kumar et al. [9], we hypothesized that the dissection procedure could have influenced the results because the neurovascular bundle reaching the muscle had to be dissected, including the careful removal of the muscle’s fascia, so that the course from the NTIO’s subdivisions reaching the posterior border of the inferior oblique muscle could be determined precisely. Both muscle surfaces (superior–ocular and inferior–orbital) should be carefully assessed after the muscle is cut from its origin (Figure 3).

The existing literature lacks a histological examination of the interface of the neurovascular bundle and the posterior border of the inferior oblique muscle, including the NTIO’s entry point to the muscle’s belly. The results presented in this study show a histological analysis of the angulation of the muscle’s entry point of the nerve to the inferior oblique muscle. These data complement previous works and demonstrate the curved course of the NTIO’s muscular sub-branches at the level of their origin (just prior to reaching the inferior oblique muscle’s posterior border).

Ana-Magadia et al. [11] measured the horizontal distance between the origin of the inferior oblique muscle and its junction with the NTIO (origin–junction distance) and estimated it at 10.6 mm (±3.6 mm). The same authors measured the vertical distance between the NTIO’s entry to the muscle’s posterior border and the inferior orbital rim (floor– or rim–junction distance) in cadavers; on average, over the entire sample, the distance was 3.4 mm (±1 mm). In our study, the analogous vertical dimension (rim–junction) was similar, estimated at 3.66 mm (median = 3.21 mm, S.D. = 0.93 mm). Tsutsumi et al. [10] examined magnetic resonance images in the sagittal and axial planes and found that the mean distance from the orbital floor to the inferior oblique muscle’s innervation site (NTIO’s entry point) was 3.9 mm (range 1.7–8.7 mm) on the right side and 4.1 mm (range 1.4–6.5 mm) on the left. As Ana-Magadia et al. [11] concluded, such landmarks, as the origin of the inferior oblique muscle and the inferior orbital rim, can be practical and reliable reference points for predicting the location of the NTIO’s entry to the belly of the inferior oblique muscle during a posterior–inferior orbitotomy, irrespective of the sex.

Other landmarks can also be used to determine the point of entry of the NTIO to the inferior oblique muscle. Gupta et al. [13] assessed the distance between the muscle’s origin and the nerve’s entry point. They found that the NTIO was 28 mm long, and the mean distance of the nerve’s entry point to the muscle’s origin was 15.5 ± 2.3 mm. Our value for the mean NTIO length was 30.6 mm (median = 29.9 mm, S.D. = 3.41 mm), and the distance from the nerve’s entry point to the inferior oblique muscle’s border was 15.23 mm on average (median = 15.06 mm, S.D. = 5.9 mm).

### 4.3. Clinical Significance

As Erdogmus et al. [8] stress, several surgical approaches can expose lesions within the orbit. During such approaches, detailed anatomical knowledge of the neurovascular structures is vital for avoiding traumatic retraction or injury to the nerves reaching the extraocular muscles. This statement also applies to the NTIO and its topography.

Knowledge of the anatomy of the inferior oblique muscle and its motor branch (NTIO) is vital during ocular and craniofacial surgeries [10,11,24,25,26,27,28]. Also, those structures have to be kept in mind when a safe intervention via lateral and transmaxillary routes is attempted [10,25,28]. As Tsutsumi et al. [10] stress, the NTIO traverses the inferolateral area of the surgical field close to the orbital floor and can be injured during a lateral orbitotomy. The nerve exits from the inferior division of the oculomotor nerve near the lateral border of the inferior rectus muscle and, during its further course, remains in complex anatomical and topographical relationships with the orbital fat pad, connective tissues, and the pulley system of the inferior oblique muscle. In the distal part, it forms an upward angulation before reaching the belly of the inferior oblique muscle and finally entering it. The relationship between the NTIO, orbital connective tissues, and the pulley system should be kept in mind by surgeons, as they will need to navigate these structures during procedures [10]. Understanding these anatomical relationships is also crucial when assessing clinical outcomes of orbital floor fractures, as modern MRI studies have highlighted the significance of these connective tissues in the functional anatomy of the inferior oblique and inferior rectus muscles. Takahashi et al. [7] found that the NTIO was trapped in 18.6% of the patients suffering from an orbital floor trapdoor fracture with orbital fat incarceration. Inferior oblique muscle paresis, mydriasis, and accommodative palsy have also been described as temporary complications of sinus surgery [29].

A study by Tsutsumi et al. [10] was a pioneering trial of MR imaging exclusively focused on the NTIO. It is worth mentioning that modern imaging diagnostics enable increasingly accurate imaging of anatomical structures to be obtained and are used in diagnosis and treatment.

### 4.4. The Neurofibrovascular Bundle’s Relation to the Ancillary Origin of the Inferior Oblique Muscle

The functional anatomy of the inferior oblique muscle, particularly its positional changes during gazing, is closely linked to the connective tissue pulley system, which stabilizes and directs the muscle’s action. The characteristic morphology of the distal NTIO part may be influenced by specific biomechanical conditions within the orbit. Contrary to Stager’s [24] proposal that ‘the neurofibrovascular bundle of the inferior oblique muscle possesses ligamentous qualities that enable it to function as an ancillary origin for the muscle’, our observations suggest that the NTIO alone lacks sufficient connective tissue support to transmit the forces required to serve as an ancillary origin for the inferior oblique muscle. Stidham et al. [30] also explored the stiffness of the NTIO’s neurofibrovascular bundle, highlighting its role in maintaining muscle alignment and tension during movement.

However, current anatomical knowledge suggests that a robust connective tissue pulley system surrounds both the inferior rectus and inferior oblique muscles at the NTIO’s entry point, providing essential stabilization and mechanical support. It is crucial to emphasize that this pulley system, rather than the neurovascular bundle itself, functions as the true ancillary origin, reflecting the anatomical and topographical relationships between the NTIO and the pulley system, rather than a direct biomechanical influence of the NTIO’s neurovascular bundle on the muscle function.

Although the NTIO and neurovascular bundle are topographically closely associated with this pulley, it is the pulley that plays a pivotal role in directing the forces generated during muscle contraction. This connective tissue network not only anchors the muscles but also coordinates their movement by guiding the paths of nerves and vessels. The NTIO’s positioning within this complex environment suggests that its characteristic arcuate course (angulation) just before entering the muscle’s belly may be an adaptive feature. This angulation likely allows for the NTIO to withstand varying mechanical stresses and aligns its trajectory with the dynamic movements of the inferior oblique muscle, thereby reducing potential tension and minimizing the risk of nerve damage during muscle contraction. This angulation, combined with the loose connection to the pulley between the inferior oblique and inferior rectus muscles, enables the neurovascular elements associated with the inferior oblique muscle to adapt effectively to the biomechanical demands of ocular movements.

### 4.5. Study Limitations

Our study has several limitations. First, the use of classical dissection methods did not allow for a detailed visualization of the segregation of the NTIO’s muscular sub-branches within the inferior oblique muscle’s belly. Although selective projections of the motor sub-branches could be more accurately examined using Sihler’s staining technique, we were unable to utilize this method because of technical difficulties in the current study. Similar research can be supplemented with Sihler’s stain for each type of NTIO entry to the inferior oblique muscle’s belly to expose the intramuscular distribution of motor sub-branches. As Shin et al. [12] stress, Sihler’s staining helps to expose the gross nerve distribution in the inferior oblique muscle. It has become very popular during the past decade and is used to examine various muscles and other anatomical structures in human and animal subjects [14,20,31,32,33]. However, this limitation does not affect the practical value of this work because anatomical structures are localized macroscopically or on the basis of anatomical landmarks during surgical procedures.

Second, the complete removal of the connective tissue system of the orbit, including the pulleys of the inferior oblique and inferior rectus muscles, restricted our ability to analyze the relationship between the NTIO and these critical structures. We recognize that the concept of muscle pulleys and their functional role cannot be fully addressed when connective tissue structures are removed, as this approach inherently simplifies the anatomy to facilitate gross examination. More detailed studies could explore the topographical anatomy of the NTIO in relation to the orbital pulleys without removing fascial structures, providing a more comprehensive understanding of these complex anatomical relationships.

Future studies should incorporate advanced staining techniques and the preservation of orbital connective tissues to better address both above-mentioned limitations. However, this study complements previous data with a detailed analysis of the junction between the inferior oblique muscle’s belly and its motor branch (NTIO) subdivisions.

## 5. Conclusions

### 5.1. Anatomical Variations of NTIO Entries

Three distinct types of NTIO entries to the inferior oblique muscle’s belly were identified. The most common type involved the NTIO entering the muscle’s inferior (orbital) surface, while the less-common types involved entry to the superior (ocular) surface or a split distribution across both surfaces.

### 5.2. Histological Findings

Histological examination confirmed that the distal part of the NTIO exhibits a characteristic arcuate course (angulation) before reaching the muscle’s belly. Histologically, the NTIO’s neurovascular bundle itself lacks the mechanical properties needed to function as an ancillary origin.

### 5.3. Clinical Implications

Understanding the precise anatomical variations and the distribution of the NTIO’s motor sub-branches can enhance surgical approaches and the management of ocular motor disorders.

## Figures and Tables

**Table 1:** Morphometric measurements of the inferior oblique muscle (IOM) and its motor branch (nerve to the inferior oblique muscle—NTIO).

	NTIO Diameter	NTIO Length	IOM Origin Width	IOM Origin Thickness	IOM Insertion Width	IOM Length	NTIO’s Entry—Distance from IOM Origin	NTIO’s Entry—Distance from Inferior Orbital Rim
Min.	0.61	25.25	2.66	0.97	6.26	28.03	11.8	2.87
Max.	1.05	38.26	4.83	1.88	9.08	33.92	19.6	6.27
Mean	0.82	30.6	3.29	1.39	7.76	31.2	15.23	3.66
Median	0.82	29.9	3.04	1.36	7.84	31.3	15.06	3.21
S.D.	0.12	3.41	0.58	0.26	0.79	1.72	2.09	0.93

## Data Availability

The data presented in this study are contained in the paper.
